# Ritualized ink use during visual courtship display by males of the sexually dimorphic cuttlefish *Sepia andreana*

**DOI:** 10.1002/ece3.10852

**Published:** 2024-02-02

**Authors:** Arata Nakayama, Shunsuke Momoi, Noriyosi Sato, Tomohiko Kawamura, Yoko Iwata

**Affiliations:** ^1^ Atmosphere and Ocean Research Institute The University of Tokyo Kashiwa Chiba Japan; ^2^ Aquarium Asamushi Aomori Japan; ^3^ School of Marine Science and Technology Tokai University Shimizu Shizuoka Japan

**Keywords:** Cephalopoda, cuttlefish, ink, reproductive behavior, sexual dimorphism

## Abstract

Visual display is a crucial aspect of courtship, and their success relies on both display quality and the surrounding environment, such as the visual background. Cephalopods may release ink when attacked by predators or during aggressive interactions with conspecifics. Here, we report that ink is used as a part of the courtship display by males of the cuttlefish species *Sepia andreana*. Males of this species engage in a highly ritualized multimodal courtship using a pair of markedly long sexually dimorphic arms. At the climax of the courtship, the male releases a diffuse backdrop of ink near himself and then performs the specific courtship display by extending his sexually dimorphic arms and altering his body pattern to pale in front of this ink backdrop, and then proceeds to mate. This novel use of cephalopod ink could make the surroundings darker and more homogeneous, potentially serving as a temporary modification of the visual environment for courtship display.

## Graphical abstract

Cephalopods may release ink when attacked by predators, but we have discovered that ink is used as a part of the courtship display by males of the cuttlefish species *Sepia andreana*. Males of this species engage in a highly ritualized multi‐modal courtship using a pair of markedly long sexually dimorphic arms, and at the climax of the courtship, the male releases a diffuse backdrop of ink near himself and then performs the specific courtship display in front of this ink backdrop. This novel use of cephalopod ink could potentially serve as a temporary modification of the visual environment for courtship display.

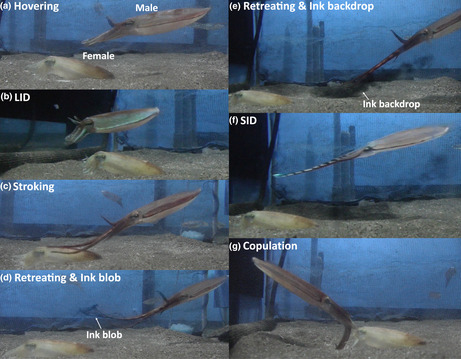

## 1 INTRODUCTION

Visual display is an essential component of courtship, the success of which depends on both display quality and the surrounding environment, including visual background or threats by rival mates or predators. Therefore, some animals choose a particular display environment (Cole & Endler, 2016; Endler et al., 2022) or even modify it, as do bowerbirds, for example (Clifford & Dawn, 2004). Such modification attracts female attention prior to the courtship display (Borgia, 1995), visually emphasizes the courting male (Endler et al., 2010), and can reduce the risk of predation (Cestari & Pizo, 2014). However, modifying the courtship site burdens the male with significant time and energy costs (Laidre, 2021) and then constrains him to perform his display only at that particular site (Schaedelin & Taborsky, 2009).

Cephalopods have a complex brain and apparently high cognitive ability (Darmaillacq et al., 2014), which enable complex intra‐specific visual communication using instantaneous changes in body pattern, skin texture, and posture (e.g., Jantzen & Havenhand, 2003). Their body pattern plays a significant role in reproductive communication, and when signaling to conspecifics, they chose a visually less complex, homogeneous background to enhance the contrast of their display against the background (Zylinski et al., 2011). Cephalopods may release ink when attacked by predators or during aggressive interactions with conspecifics (Allen et al., 2017). Here, we report that ink is used as a part of the courtship display by males of the cuttlefish species *Sepia andreana*.

**FIGURE 1:**
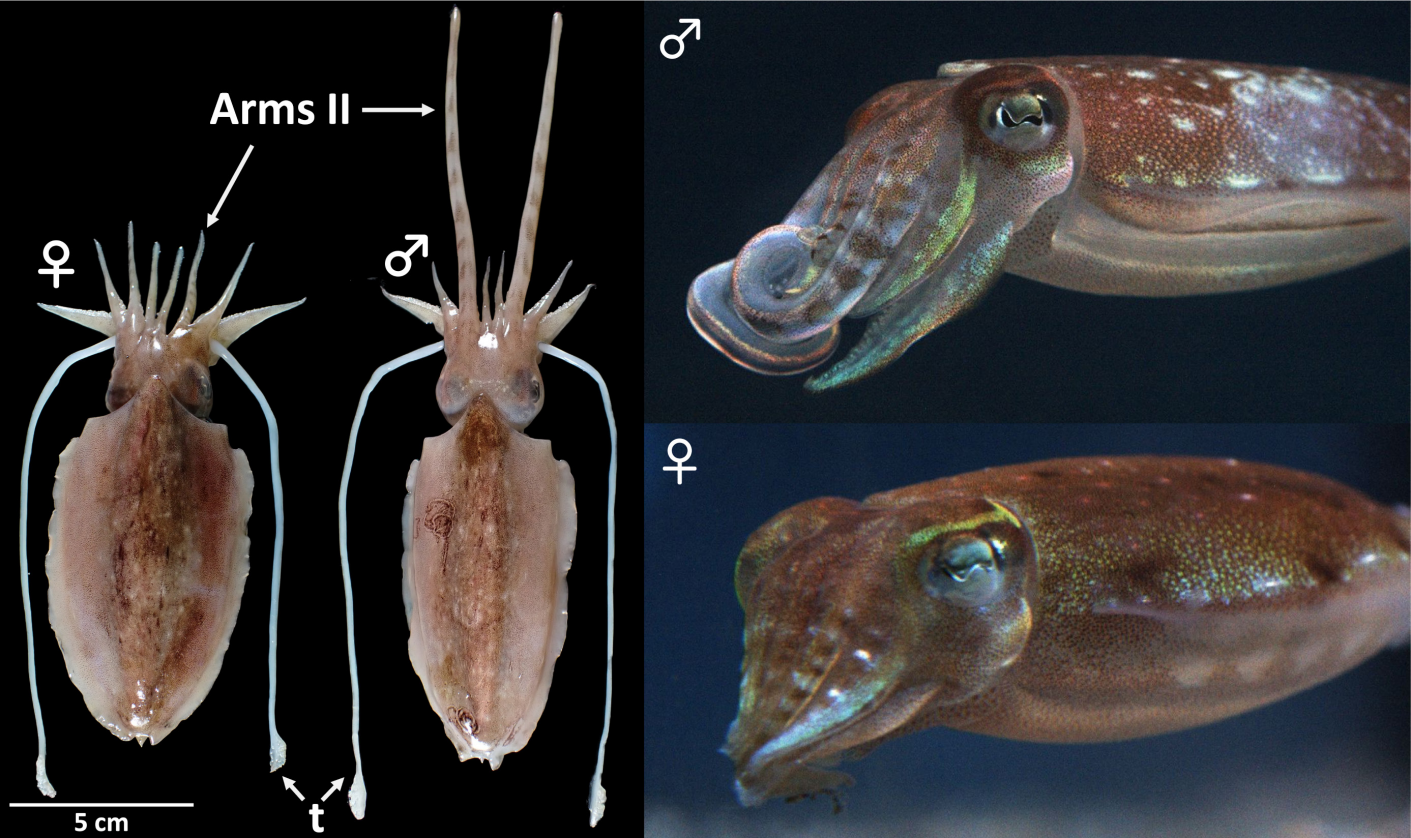
Sexual dimorphism in *Sepia andreana*. The second pair of arms (“arms II”), counting from the dorsal midline, is very much longer and stouter than the others in male. “t,” tentacles, which in living cuttlefish are not usually visible, are retracted into pockets beneath the head unless ejected to catch prey.

*Sepia andreana* has a sexually dimorphic pair of arms (arms II), which, in the male, is markedly modified: these arms are very much longer and stouter than the others with a bluntly rounded distal end (Figure 1), and the male mantle and cuttlebone are slightly narrower than in the female (Sasaki, 1929). This species belongs to the group of *Doratosepion* species complex (Lupše et al., 2023). This group, characterized by a narrow cuttlebone (Khromov et al., 1998; Roeleveld, 1972), is supposed to be monophyletic (Lupše et al., 2023; Yoshida et al., 2006), and contains approximately 43 species among a total of 114 nominal sepiid species (Ho & Lu, 2005; Reid, 2000). Some *Doratosepion* species have remarkable secondary sexual traits, such as extremely long arms and enlarged fins (Reid et al., 2005). Despite their characteristic sexual dimorphism and species richness, no study has yet described their reproductive behavior. This study conducted detailed observations of the reproductive behavior of *S. andreana*, revealing two previously unknown forms of cuttlefish courtship display: (1) use of the male elongated arms II to alter visual appearance and to make physical contact with the female; (2) use of the ink as additional cues. We propose that the use of ink during courtship display may serve as a cost‐effective way to rapidly change the visual environment to advertise himself to attract female attention.

## 2 MATERIALS AND METHODS

### 2.1 Field collection and maintenance of cuttlefish

Mature specimens of *S. andreana* (approximately 70–100‐mm mantle length) were collected from an inshore set net (a static, anchored net enclosure with a funnel‐like entrance, capturing fish passively when they swim into it by chance) off the coast of Tairadate (Aomori, Honshu, Japan). They were captured during February–March 2020–2023 and transported in a 2000‐L tank of fresh seawater aerated with O_2_ to Asamushi Aquarium, Aomori, Japan. Males and females were kept in two tanks (134 × 90 × 70 cm) separately using a filtered recirculating water system. The water flow in the aquarium was approximately 0.03 m s^−1^. Water temperature was monitored continuously and maintained within the range of 14–16°C, which is within the normal temperature range for this species. Photoperiod (9L:15D) was maintained using white fluorescent lights during the day from 08:00 to 17:00. The cuttlefish were fed Pacific krill (*Euphausia pacifica*) once daily. Some males were kept in an exhibition tank at the aquarium and moved into an observation tank, ready for behavioral observations. All procedures performed in the present study followed the ethical standards of the Life Science Research Ethics and Safety Committee of the University of Tokyo and were approved by the committee (Approval number: P20‐7).

### 2.2 Observation procedures

Behavioral observations were conducted during February–March from 2020 to 2023. Cuttlefish were transported from the maintenance tanks to an observation tank (180 × 120 × 90 cm). During experiments conducted in 2023, a partition was placed down in the middle of the tank, allowing for two sets of observations to be made simultaneously. The observation tank, containing filtered recirculating seawater, was cordoned off from other parts of the building to prevent disturbance from other visual stimuli and was lit during “daylight” hours (08:00–17:00) with a white LED light.

After 1 h of acclimation to the observation tank, behavioral observations and recordings were conducted between 09:00 and 17:00 with two digital video cameras. One was fixed in place so as to continuously record reproductive behaviors occurring anywhere within the tank, while the other was a hand‐held camera used to zoom in and capture each phase of the reproductive behaviors. The fixed camera was positioned on one side of the tank, and the hand‐held camera was moved to various positions as required to follow the reproductive behaviors. A total of six observations using multiple males in each (multiple males trial; 2–5 males with 1–5 females) and 30 observations using a single male in each (single male trial: 1 male and 1–5 females) were conducted using 13 males and 43 females in total. Due to the limitation of available individuals, especially males, some individuals were used several times in combination with different partners. During courtship in *S. andreana*, 11 distinct behavioral components specific to courtship were identified (see Section 3). Therefore, we defined the beginning of courtship as the first observed instance of any of these behavioral components. For each male, the probability of transitioning from one behavioral component to another was calculated. The means of the probabilities for all individuals were calculated and used to create a transition diagram. If the courtship behavior was not filmed from the beginning, the probability was calculated using only the behavioral components observed. Since two types of courtship behavior were identified (see Section 3), the success rate of courtship was compared between the two types by Fisher's exact test.

### 2.3 Measurement of dried ink sac weight

For morphological measurements, freshly dead specimens of mature *S. andreana* (from the same source as that from which live animals were obtained) were stored in a freezer for a few months until processing. The weight of the dried ink sac, without its contents, was used as an indicator of ink sac size (because cuttlefish often release ink during the trauma of capture, thus rendering inaccurate any measurement of the exact amount of ink contained within). After measuring body size and dissecting out the ink sac from each individual, the contents of the ink sac were removed, and the empty sac was dried at 60°C for 12 h before weighing. Analysis of covariance (ANCOVA) was performed with the dried ink sac weight as the dependent variable, sex as the covariate, and head width as the independent variable. Although mantle length is commonly used as an index of cuttlefish body size, head width was chosen because the relative proportions of mantle and head shape in *S. andreana* were found to be clearly sexually dimorphic, with males being relatively more slender than females (ANCOVA, mantle width as a dependent variable, mantle length as an independent variable, sex as a covariate, *F* (1, 86) = 79.74, *p* < .01). We confirmed that our data met assumptions for ANCOVA by examining the residuals for normality (Shapiro–Wilk test, *W* = 0.98, *p* = .24) and homoscedasticity (Bartlett's test, *K*‐squared = 1.5, *p* = .22). All statistical tests were performed with R version 4.1.2 (R Core Team, 2021).

### 2.4 Measurement of light absorbance by ink

Ink was retrieved from the ink sacs of seven males that had been used for behavioral observations and had died naturally in the aquarium without releasing ink, which was removed within the intact ink sac shortly after death and promptly frozen at −40°C to minimize any deterioration. The ink was removed from the ink sac, weighed, and serially diluted with seawater to make aqueous suspensions with final concentrations of 0, 9.1, 16.7, 23.1, 28.6, 33.3, 37.5, and 41.2 mg L^−1^. These ink suspensions were mixed to homogeneity and poured into a plastic container (5 × 10 × 10 cm). An LED light (79.4 lux) was placed parallel to the side of the container to illuminate the ink solution, and the amount of light passing through 10 cm of this solution (estimated as equivalent to the approximate thickness of the ink backdrop released by males during their courtship display) was captured with a digital camera (TRI050S‐P/Q, LUCID). This camera allows manual adjustments of aperture and shutter speed, with a constant ISO value maintained throughout the experiment. The intensity of the transmitted light was measured by ImageJ (NIH, version 1.53f), and the relative intensity to that of light transmitted through control (inkless) seawater (0 mg L^−1^) was calculated.

## 3 RESULTS

Aquarium‐based observations (36 courtship events) revealed that males conduct a ritualized courtship involving specific displays with their sexually dimorphic arms, accompanied by two apparently novel types of ink release, for each of which the ink seems to be distinct in both amount and viscosity.

**FIGURE 2:**
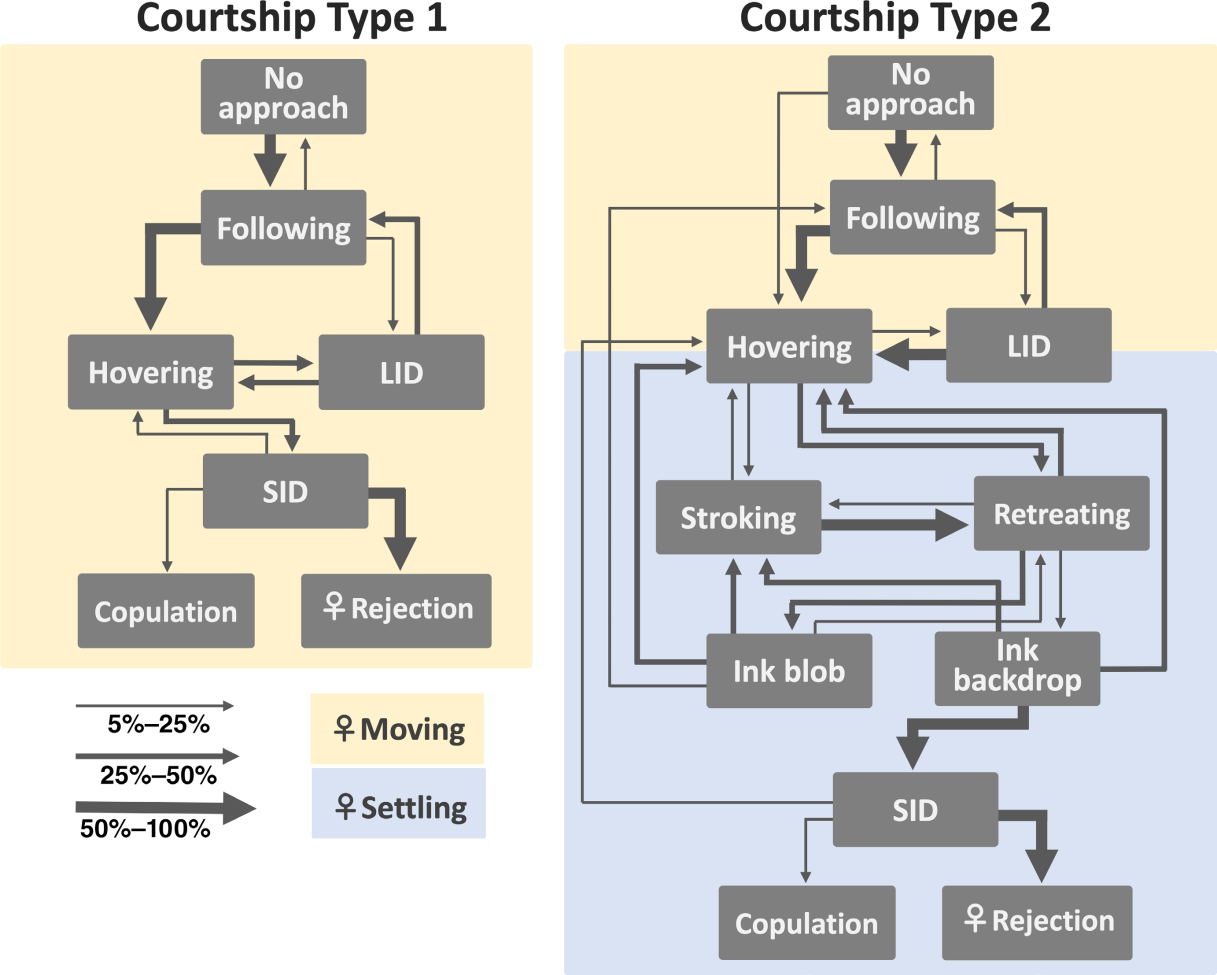
Flowchart of behavioral transition probabilities in the two types of courtship of *Sepia andreana* based on observations of Type‐1 (*n* = 19) and Type‐2 (*n* = 17) courtships. This flowchart refers only to male behavior, except Copulation and Rejection. The thickness of the arrows denotes the conditional probabilities of a particular transition occurring between two behaviors. Transitions with a value less than 0.05 are not included to enhance the clarity of the figures. The color of the background represents the condition of the female, which is either moving (yellow) or settling (blue).

There are two patterns of courtship (Figure 2): Courtship Type 1 and Courtship Type 2. In Courtship Type 1 (19 events by 13 males, Figure 2, Video 1), the male follows a female (“Following”) and hovers above her (“Hovering”) for a few seconds. He then shows “Stretched iridescent display (SID)” in which he performs an exaggerated elongation of the body and arms while displaying iridescent coloration on the body, a dark stripe leading from the lateral mantle to arm III, and a pattern of dark bands across his extended, long, sexually dimorphic arm II. The chromatic stripe and bands are exhibited only on the side toward the female.

**VIDEO 1:** Courtship Type 1 and Type 2 in *Sepia andreana* viewed in the observation tank at Asamushi Aquarium.

**FIGURE 3:**
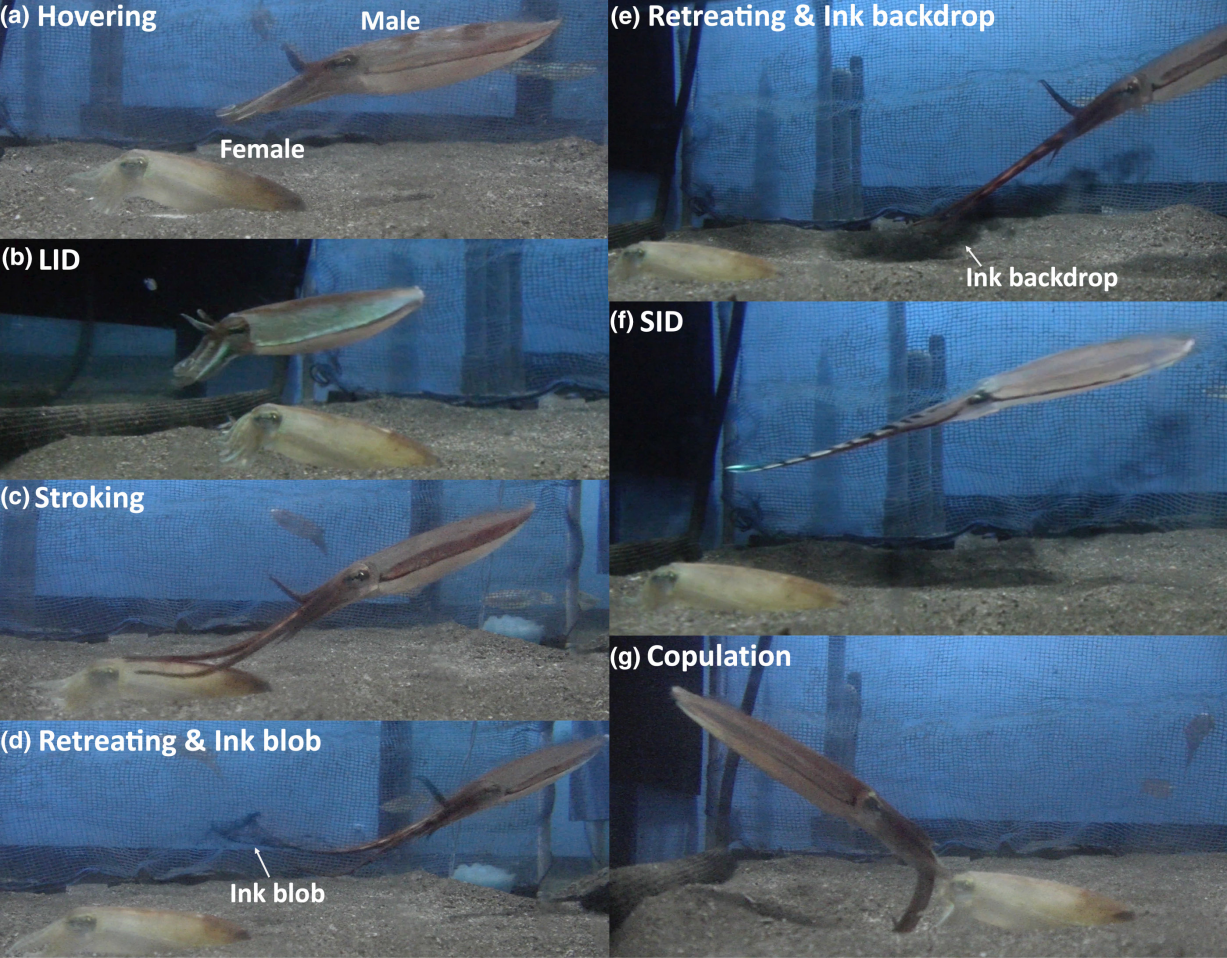
Behavioral components of Courtship Type 2. (a) Hovering. (b) Lateral Iridescent Display (LID). (c) Stroking. (d) Retreating and Ink blob. (e) Retreating and Ink backdrop. (f) Stretched Iridescent Display (SID); The iridescent coloration is strong, especially on the sexually dimorphic arms and the “eyebrow.” A chromatic dark stripe, from the lateral mantle to arm III, and the dark eyespot are also shown to the female. Ink backdrop can be seen expanding below and behind the male. (g) Mating.

**FIGURE 4:**
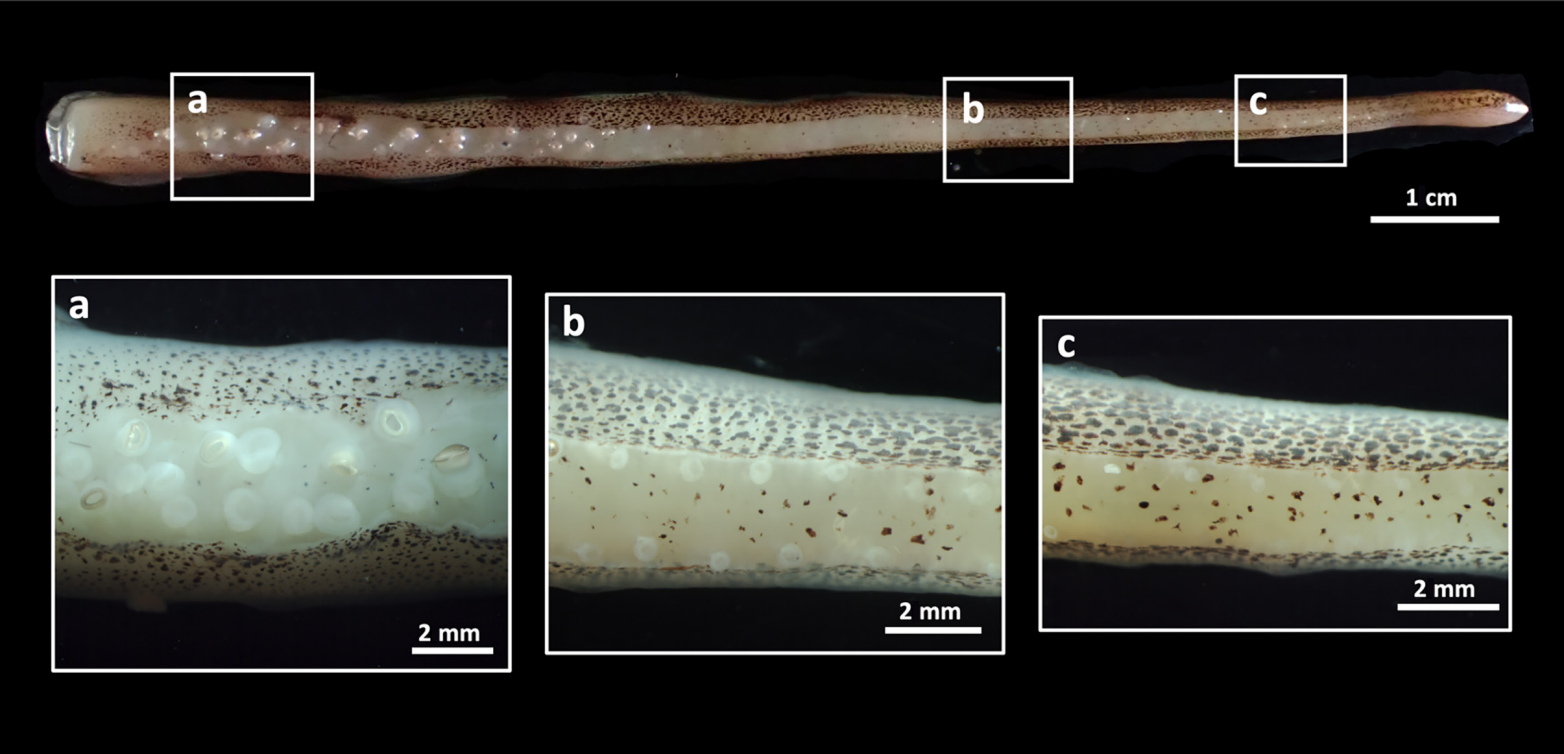
The sucker arrangement on the male sexually‐dimorphic arm II. Suckers (rounded, faint‐white profiles) on the distal half of the arms (“b,” “c”) are rudimentary and sparse (compare with the proximal, normal suckers in “a”).

Compared to Type 1, Type 2 has a long and complex behavioral sequence and is conducted toward females settled on the bottom. In Courtship Type 2 (17 events by 13 males, Figures 2 and 3, Video 1), after Following, the male induces the female to settle on the substrate by Hovering (Figure 3a) and directing the funnel to puff water jets over her. He occasionally passes diagonally over the female from her posterior to anterior and shows “Lateral iridescent display (LID, Figure 3b),” in which his roll‐upped sexually dimorphic arms (arms II), arms III, and the base of the fin only on the side toward the female reflect white or blue structural colors, probably produced by iridophores. He then extends out his sexually dimorphic arms and strokes her mantle and head using the distal half of each of these arms (“Stroking,” Figure 3c). Suckers on the distal half of the male's sexually dimorphic arm are rudimentary and sparse (Figure 4), probably in relation to the Stroking.

**FIGURE 5:**
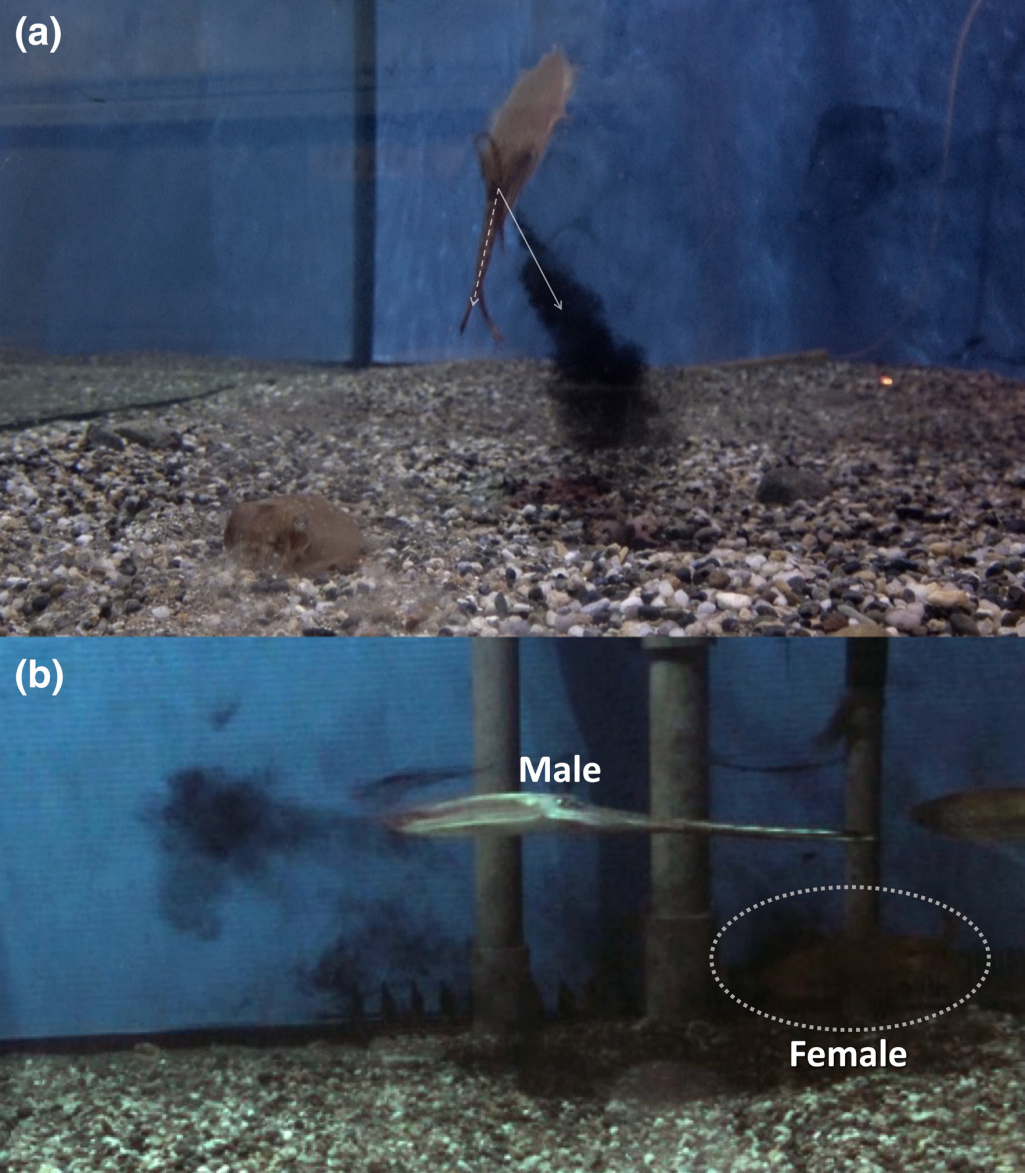
Ink backdrop and SID seen from different angle. (a) Front view of the male discharging Ink backdrop to his side directed away from the female by directing the funnel ventrolaterally just before SID. Straight and dashed arrows indicate the angle of ink release and the orientation of the male's midline, respectively. (b) SID seen from behind the male, with the Ink backdrop spreading and hiding the courted female from view. The dashed circle indicates the position of the courted female.

**FIGURE 6:**
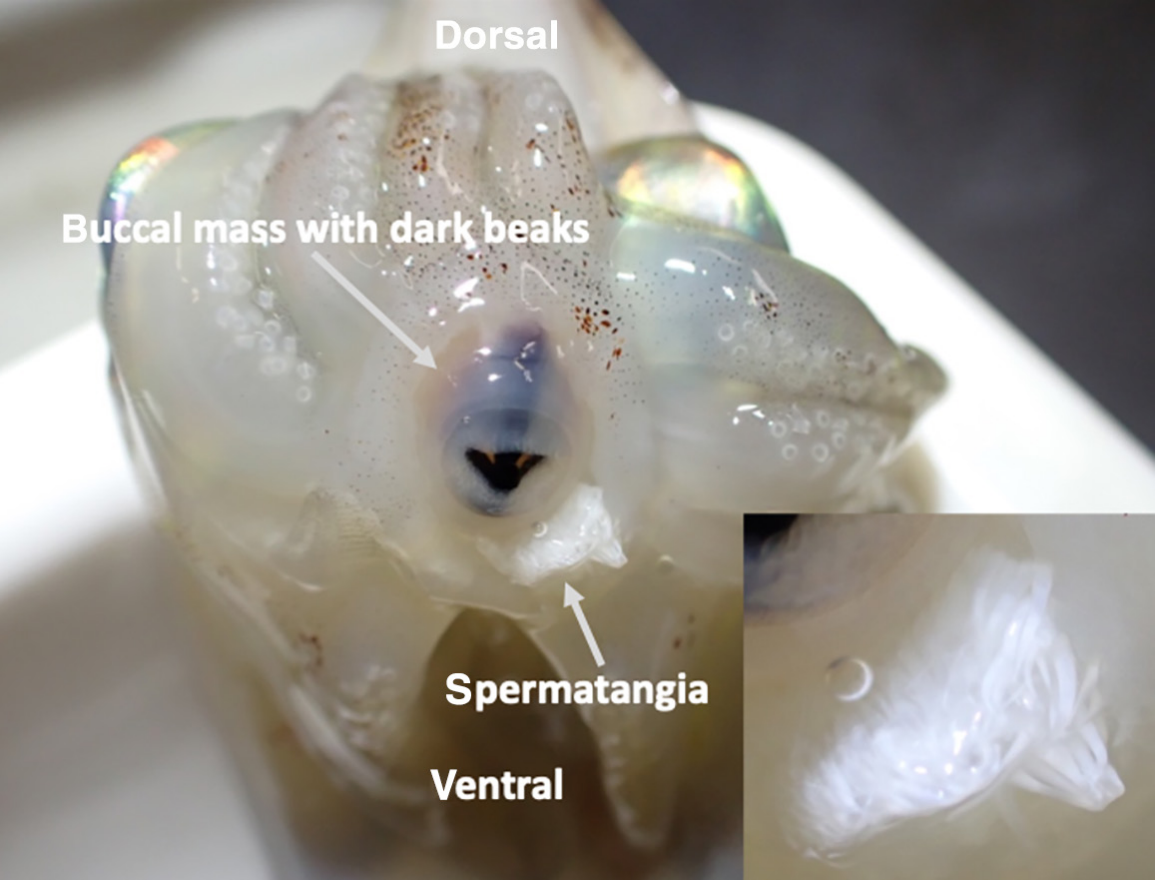
Bundle of spermatangia attached around the buccal mass of the female (inset at higher magnification showing the many individual spermatangia). Each spermatangium is one spermatophore that has everted upon implantation in the buccal mass of the female. The sperm inside will be used later for fertilization at the time of egg laying by the female.

His pair of arms I, the first pair of arms counting from the dorsal midline, are held up above the level of his head. Bands of darkened chromatophores are displayed on the sides of his specialized long arms, but iridescent structural reflection is not apparent except at the ends of these long arms. A dark line is displayed on the lateral mantle, just under the fin. During Stroking the male periodically retreats and swims behind the female with his sexually dimorphic arms extended but no longer in contact with the female (“Retreating,” Figure 3d). When Retreating, he ejects a small ink blob that is smaller than his head and remains suspended above the female for a while (“Ink blob,” Figure 3d). After Hovering, LID, Stroking, and Retreating, the male releases ink of a different consistency (“Ink backdrop”) which is similar size as his entire body and soon diffuses near the male on the side facing away from the female (Figures 3e and 5a). After releasing the ink backdrop two or three times serially, he then places himself between this backdrop and the female and shows a SID (Figures 3f and 5b). In both types of courtship, mating exclusively occurs immediately following the SID (Figure 3g). The female opens her arms while the male attaches spermatophores around her buccal mass with the hectocotylus (left arm IV) in head‐to‐head position for only about 1 s (see Figure 6 for spermatangia attached around the female buccal mass). When a female does not accept the courtship, she either remains in place without reacting (following which the male may have another attempt at courtship) or completely rejects it by jetting rapidly away (Video 1). The probability of successful mating was noticeably low: one out of 19 events of Courtship Type 1 and two out of 17 events of Courtship Type 2 resulted in successful matings. There was no significant difference in the probability of successful copulation between Type 1 and Type 2 (Fisher's test, *p* = .60).

**FIGURE 7:**
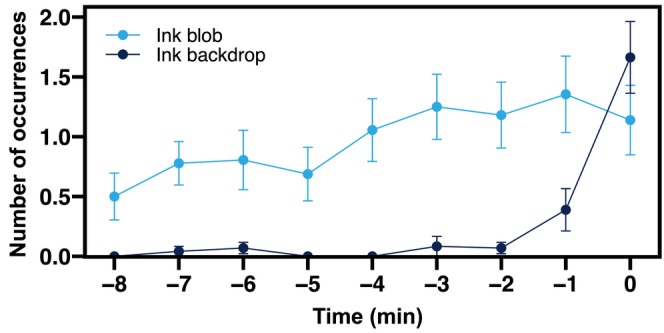
Mean frequency (times min^−1^) and timing of the two kinds of inking behavior observed during the 9 min prior to the SID. Time 0 indicates the time when SID begins. Each plot indicates the mean number of instances of observed ink release during the time period (*N* = 12, mean ± SE). Blue: Ink blob; Black: Ink backdrop.

While ink blobs were released repeatedly during Stroking, most ink backdrops were released within a minute before commencing the SID (Figure 7). Although the SID itself was similar for both Type 1 and Type 2 courtship sequences, ink backdrop was observed almost exclusively in Courtship Type 2 (1 in 19 events in Courtship Type 1, 17 in 18 events in Courtship Type 2, Figure 2). Courtship Type 2 is usually conducted after Type 1, but may proceed without it. The full sequence of Courtship Type 2 takes a relatively long time (50.5 ± 21.3 min, mean ± SD, time taken from initial approach to SID without interruption) compared to Courtship Type 1 (1.6 ± 2.8 min). The behavioral sequences were clearly categorized, suggesting that they are not unusual behaviors occurring under artificial aquarium conditions but ritualized courtship behaviors (Figure 2).

**FIGURE 8:**
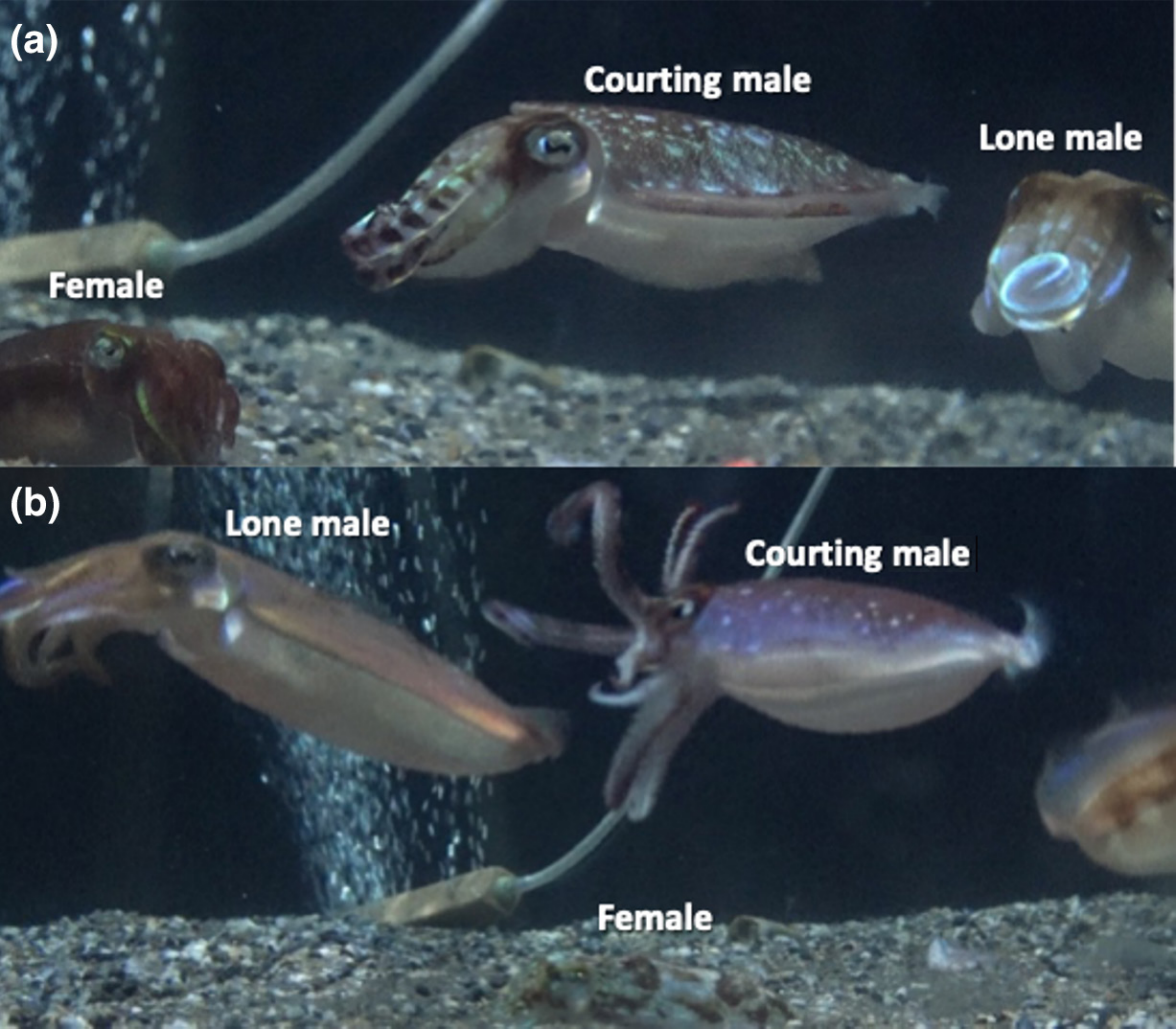
Agonistic behavior in male *Sepia andreana*. (a) Agonistic display shown by the courting male (center). To the right of him is another male, and to the left is the courted female. (b) Courting male attempting to grab lone male. To the left of him is another male; under him, is the courted female.

While observed courtship procedures were not different between the Multiple males trials and Single male trials, during Courtship Type 2 in Multiple males trials, the courting male occasionally conducted aggressive behavior in which he dashed toward a nearby male with an Agonistic Display in which dark rings are exhibited around his eyes, his long arms II are still rolled up but show the banding pattern more clearly than usual, and the mantle has a speckled pale pattern (Figure 8). Dark eye ring is reported also for fights among males of other cuttlefish species (Allen et al., 2017). In some instances, the courting male attempted to grab the other male with his arms and bite him (Video 2). SID was not shown in these situations. In all cases, the attacked male did not show Agonistic Display in return nor fight back and merely fled.

**VIDEO 2:** Attacking behavior in *Sepia andreana*. The courting male rushes to the nearby lone male and attempts to bite him. The attacked male flees and does not approach again.

**FIGURE 9:**
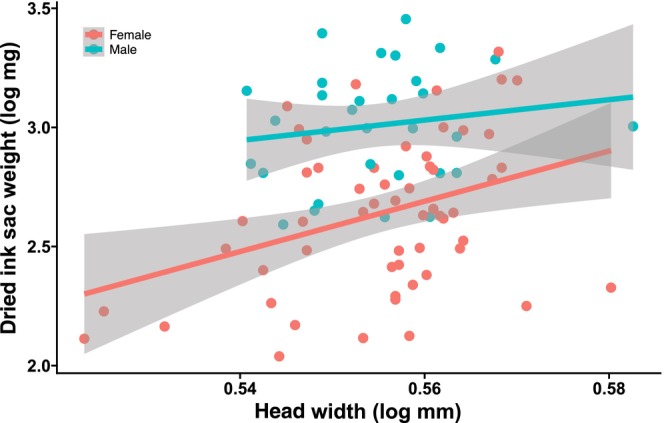
Allometric relationship between head width (as an index of body size) and dried ink sac weight.

**FIGURE 10:**
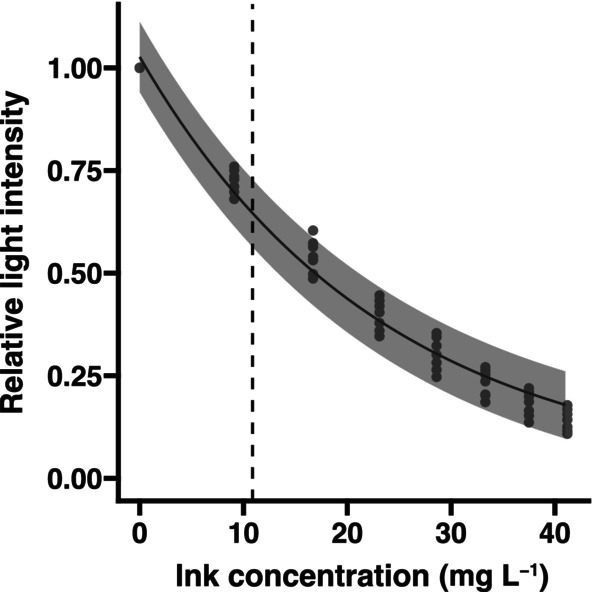
Relationship between ink concentration and the relative intensity of transmitted light. The intensity of LED light passing through each ink solution at a standard thickness of 10 cm (estimated approximate depth of the ink backdrop) was measured relative to the intensity of light passing through plain seawater (zero, at left). Shading: zone of 95% confidence. Broken line: estimated approximate concentration of the ink backdrop released by a male under the assumption that the male releases 1/50 of the mean weight of ink sac contents into 2 L of seawater.

Measurement of ink sac weight, with its contents excluded, showed that males have a relatively heavier ink sac than females (ANCOVA; *F* (1, 86) = 36.46, *p <* .01, Figure 9). In addition, the standard ink solution that resembled Ink backdrop demonstrated efficient light absorption (Figure 10).

## 4 DISCUSSION

Cephalopod ink is a mixture of two components from different organs: a melanin‐containing black liquid from the ink sac; and a mucous secretion assumed to be produced by the funnel organ (Derby, 2014). Variations of this mixture produce inks of different consistency, such as a diffuse cloud used as a smokescreen to hide the inking individual, or a discrete blob ejected as a decoy to distract the attention of a predator (Derby, 2014). During the courtship by *S. andreana*, two types of ink, ink blob and ink backdrop, were released. These types of ink were apparently distinct from each other in form, amount, and timing of release, suggesting that each has been co‐opted for a purpose different from typical usage. Ink blobs released repeatedly during Stroking seem to contain a relatively large amount of mucus, slowing dispersion and allowing the ink to remain suspended as a viscous blob above the female for a while. Cephalopod ink serves as an intra‐specific visual alert, evoking cryptic behavior in some cephalopods (Wood et al., 2008), so the ink blobs suspended above the courted female may induce her to remain close to the substrate, which is a primary defense behavior of some squid and cuttlefish (Staudinger et al., 2011).

The property of the ink blob (i.e., long‐lasting use of a small amount of ink) is suitable for an extended visual‐alert effect. However, the ink backdrop released immediately before SID is apparently less viscous than a blob, containing almost no mucus and spreading widely and quickly. Given that the dispersed ink of *S. andreana* is efficient at absorbing ambient light, this ink backdrop is optimal for creating a blanket visual effect quickly and may serve to attract the female's attention to the male's display, or possibly to hide the female from rival males. In addition, this widely‐ and evenly‐diffusing ink can function as an instant backdrop against which the male can perform SID effectively. The recognition of a visual signal relies heavily on the contrast in both intensity and complexity between the signal and the background (Hailman, 1977). Consistent with that, cuttlefish tend to choose less complex backgrounds when signaling to conspecifics (Zylinski et al., 2011). Presumably, ink backdrop has two functions: reducing the brightness of the surroundings and reducing the contrast of the various structures and substrates behind and below the male (making these components more homogeneous). These both act to render the iridescent and black banding pattern on the male's sexually dimorphic arm more conspicuous for a brief period. Ink backdrop is released, directed away from the female. Cuttlefish ink is discharged from the funnel, which is a ventral, subconical tube through which water is expelled from the mantle cavity during locomotion and respiration. While cuttlefish can adjust the direction of the ink release by bending the funnel, its position in relation to the body limits where the ink can be released. Furthermore, because the funnel simultaneously controls the direction of ink release and propulsion, when the male releases ink while retreating backward for SID, the position of the ink will inevitably be in a direction roughly opposite to his movement. Male *S*. *andreana* releases ink with his funnel directed slightly away from the female, which is believed to be an adjustment of the release direction to maximize the ink's effectiveness as a backdrop against which to perform. The heavier ink sac and the resulting rich amount of ink that the male possesses could be co‐evolved with this unique courtship behavior in *S. andreana*. This proposed function of inking during courtship requires further experiments (such as experimentally manipulating the background while quantifying changes in inking behavior) to test if it could be held.

Cuttlefish are renowned for their complex reproductive interactions, yet our knowledge is limited to a few species, particularly *Sepia apama* and *Sepia officinalis*. In these species, male cuttlefish present a high‐contrast, male‐specific display called the “Zebra display,” which serves for both agonistic and sex‐recognition signals. This display may be shown during any of their encounters, and if the same display is shown in return by the other individual, that individual is recognized as a male and a fight may ensue; otherwise, that individual is perceived to be a female, and consequently, a mating attempt may ensue (Hanlon & Messenger, 2018; Tinbergen, 1939).

Recent studies on other cuttlefish species have begun to uncover a variety of sophisticated reproductive behaviors, such as a deceptive dual display in *Sepia plangon* (Brown et al., 2012) and the conspicuous courtship display of *Metasepia pfefferi* (Hanlon & McManus, 2020). The *Doratosepion* species complex encompasses nearly half of all cuttlefish species, and many of them exhibit marked sexual dimorphism, including males with elongated arms or enlarged fins (Reid et al., 2005). However, since most of the species live in relatively deep waters (Reid et al., 2005), their reproductive behaviors have largely remained unknown. The present study of the reproductive behavior of *S. andreana* provides the first description of such behavior in the *Doratosepion* species complex. The reproductive interactions of *S. andreana* significantly differ from those in other cuttlefish species observed before. The courtship behavior of male *S. andreana* is highly ritualized and time‐consuming, and at the climax, the male conducts a specific display using the unique sexually dimorphic arms II. This display (SID) is selectively targeting a female and is distinctly different from the Agonistic Display during attacking behavior, which appears to have a function similar to the intense Zebra display seen in other cuttlefish species. Considering the remarkable unique elongation of arms II in the male *S. andreana*, this distinctive display and sexually dimorphic arms with iridescent coloration are reminiscent of *Schizocosa* spider displays, which wave their long legs in courtship, or bowerbird males, which wave sticks during their displays, all expected to have developed as a result of female choice (Clifford & Dawn, 2004; Rundus et al., 2010). It is possible to speculate that all such displays may have the common function of maximizing the amount of the visual field of the female exposed to the movements of the courtship display.

Also noteworthy in the courtship of *S. andreana* is the multi‐modality of the signals. The characteristic sexually dimorphic arms provide not only visual stimulation but also tactile stimulation during Stroking. In addition to the longer and stouter form of the male sexually dimorphic arms, suckers on the distal half of the arms are rudimentary and sparse. These morphological characteristics enable males to gently stimulate a large area of female skin simultaneously. Although tactile communication in which males touch females with the tips of the arms during courtship has been reported for a few cuttlefish species (Hanlon & McManus, 2020; López Galán et al., 2020), Stroking with the unique sexually dimorphic arms of *S. andreana* would provide stimulation very different from that produced by the typical short and tapering arms of other cuttlefish species, and consequently appears to be an extreme form of a tactile courtship signal that facilitates female acceptance and enhances species recognition.

Our observations revealed no significant difference in the probability of successful copulation between the two types of courtship. This lack of difference can be attributed to the high rejection rate of females for both types of courtship. It remains uncertain whether or not the observed low probability of successful copulation in captivity accurately reflects the natural behavior of *S. andreana*. In previous observations of other cuttlefish species, the rejection rate in response to mating attempts was of a similar order of magnitude, both in the field (70% rejection of 122 mating attempts in *S. apama* (Hall & Hanlon, 2002)) and in captivity (41.5% succession of 82 mating attempts in *Sepiella japonica* (Wada et al., 2006)). Generally, cuttlefish species are known to be promiscuous (Hanlon & Messenger, 2018), a behavior wherein females lay eggs with the potential of being fertilized by several males. Although the possibility cannot be dismissed that *S. andreana* is particularly sensitive to the captive environment and, as a result, has a low mating success rate, this lower rate also implies that males have a strong chance of achieving paternity once they succeed in mating. The unique and costly courtship before mating in *S. andreana* may, therefore, be justified in the context of eventual reproductive success. Although it is unproven whether or not the highly ritualized reproductive behavior of males is necessary to increase the possibility of mating success, the fact that all matings and mating attempts were observed immediately after SID suggests that the highly conspicuous courtship display with sexually dimorphic arms is essential for reproductive success in *S. andreana*. Under a strong requirement for visual conspicuity in reproductive communication, modification of the display environment can be an additional tactic to enhance the likelihood that the ritualized courtship will enable successful mating. Ink release is a reasonable visual aid for conducting the display more effectively. Unlike a specially prepared courting stage, such as the bower of bowerbirds, environmental modification with ink is simple but can be performed impromptu, and thereby the male is able to court a female anywhere at a relatively low cost of time and energy. The flexibility of being able to adjust ink release for various situations, along with the well‐recognized high cognitive abilities of cephalopods and a demand for optimizing visual conspicuity in this species, would be feasible drivers for co‐opting modifications of anti‐predator traits into reproductive behavior.

## AUTHOR CONTRIBUTIONS

**Arata Nakayama:** Conceptualization (equal); data curation (equal); formal analysis (equal); funding acquisition (equal); investigation (equal); methodology (equal); validation (equal); visualization (equal); writing – original draft (equal). **Shunsuke Momoi:** Data curation (equal); investigation (equal); project administration (equal); resources (equal). **Noriyosi Sato:** Conceptualization (equal); funding acquisition (equal). **Tomohiko Kawamura:** Conceptualization (equal); funding acquisition (equal). **Yoko Iwata:** Conceptualization (equal); funding acquisition (equal); investigation (equal); supervision (equal); writing – review and editing (equal).

## CONFLICT OF INTEREST STATEMENT

The authors declare no conflicts of interest associated with this manuscript.

## Data Availability

The original data is available from the link https://datadryad.org/stash/share/3lAzqb3cLNfvzeD6TzSrOjUEI_0ZbVr3eEVm7‐29xuc and forthcoming on Dryad https://doi.org/10.5061/dryad.7sqv9s4wj.
